# Color-Dependent Polymerization: The Impact of Curing Time on the Conversion Degree and Microhardness of Colored Compomers

**DOI:** 10.3390/polym17152155

**Published:** 2025-08-06

**Authors:** Ozgul Carti Dorterler, Fatma Yilmaz, Ozge Tokul Olmez

**Affiliations:** 1 Department of Pediatric Dentistry, Faculty of Dentistry, Mugla Sitki Kocman University, Mugla 48000, Turkey; 2 Department of Restorative Dentistry, Faculty of Dentistry, Mugla Sitki Kocman University, Mugla 48000, Turkey; dt.fatmayilmaz@gmail.com; 3 Department of Chemistry, Faculty of Science, Mugla Sıtkı Kocman University, Mugla 48000, Turkey; ozgetokul@mu.edu.tr

**Keywords:** FTIR, conversion degree, colored compomer, microhardness

## Abstract

This study investigated the effects of color shade and curing time on the degree of conversion (DC) and microhardness of colored compomers. A total of 162 samples (81 for DC, 81 for microhardness) were prepared, with nine samples per color group (gold, blackberry, green, pink, orange, lemon, blue, silver) and for the control. Samples were subdivided into three polymerization subgroups (3 s/3200 mW/cm^2^, 10 s/1000 mW/cm^2^, 20 s/1000 mW/cm^2^). The DC was analyzed via fourier transform infrared spectroscopy (FTIR) and microhardness was measured using Vickers testing. Statistical analysis included two-way ANOVA and Spearman correlation (α = 0.05). The colored compomers demonstrated a significantly lower DC compared to the control group (*p* ≤ 0.001). Among the tested colors, green exhibited the lowest DC (33.3%), while orange showed the highest (51.0%). A significant difference in DC was observed across curing times (*p* = 0.005), with the 3 s and 20 s groups exhibiting significantly higher conversion rates than the 10 s group. Microhardness values exhibited significant variation depending on the color (*p* < 0.001). Gold compomers demonstrated the lowest microhardness, whereas silver compomers showed comparable performance with the control group (*p* = 0.154). A moderate correlation between DC and microhardness was observed overall (ρ = 0.42, *p* = 0.003). However, the observed relationships were color-dependent: orange displayed a strong positive correlation (ρ = 0.78), whereas pink revealed no meaningful association (ρ = −0.15). Color and curing time critically influence compomer performance. High-intensity short curing is viable for lighter colors, while darker colors require extended curing. Customized protocols are essential to optimize clinical outcomes in pediatric dentistry.

## 1. Introduction

Polyacid-modified compomer resins, commonly referred to as compomers, are an aesthetic restorative material used to repair teeth affected by dental caries [1]. First introduced to dentistry in the early 1990s, compomers represent a hybrid class of materials that merge the aesthetic benefits of conventional compomer resins with the fluoride-releasing capability and adhesive properties of glass ionomer cements [2]. The term “compomer” originates from a combination of these two materials’ names, with “comp” derived from compomer and “omer” from ionomer [3]. Colored compomer materials were introduced in 2003 to engage the attention of pediatric patients [4]. These materials have a composition comparable to that of traditional compomers, but they are uniquely enhanced with colored pigments and glitter particles. These additives meet safety standards set for food and cosmetic products and are deemed to be non-toxic, contributing to the material’s favorable biocompatibility. In addition, multicolored compomers maintain their capacity to release fluoride and can be recharged through the application of topical fluoride, making them especially appropriate for pediatric patients, particularly those with a high risk of developing dental caries [5,6]. Research suggests that colored compomers can serve as a motivational aid, especially for young children. Enabling patients to select the color of their restoration encourages their engagement in the dental treatment process, ultimately improving treatment outcomes [7]. During the polymerization of compomers, it is anticipated that all carbon–carbon double bonds in the monomers will react and integrate into the polymer chain. However, polymerization efficiency is influenced by multiple factors, including the composition of the resin material, the type and concentration of the solvents used, environmental conditions such as temperature and humidity, the oxygen levels in the air, the characteristics of the light curing unit, and the thickness of the resin layer. As a result, polymerization is not always fully completed, leaving behind unreacted monomers [8]. These remaining monomers, known as residual monomers, arise from incomplete polymerization. Research has indicated that the matrix components of these monomers may exhibit genotoxic, estrogenic, mutagenic, and allergic effects [9]. In photoactivated materials, the DC is directly influenced by the energy density delivered by the light curing unit, measured in J/cm^2^. Since energy density is the product of power density (mW/cm^2^) and exposure time (seconds), it is believed that comparable conversion rates can be achieved by adjusting these two parameters in different ways [10]. FTIR is a well-established and reliable technique for detecting C=C bond stretching vibrations before and after polymerization. FTIR operates by measuring the energy absorption at specific wavelengths or wavenumbers in order to analyze the chemical composition of the material being tested [11].

Microhardness is a critical parameter for evaluating the mechanical properties of dental materials, such as compomer resins, as it provides insight into their resistance to abrasion and overall durability, particularly when used in load-bearing restorative areas [12]. Surface hardness refers to a material’s resistance to indentation, and it significantly influences clinical performance. This property serves as a key indicator of whether a material possesses sufficient strength to withstand masticatory forces, resist abrasion, and endure orthodontic stresses [13]. Among the most commonly used indirect methods for evaluating the degree of polymerization are hardness tests [14]. Adequate polymerization depends on factors such as the light source, its intensity, its wavelength, its application time, and the size, position, and orientation of the light source tip, as well as the material’s color, thickness, and composition. Thanks to advancements in technology, high-powered polymerization devices are believed to enable sufficient resin polymerization in a shorter time, potentially enhancing the mechanical properties of the material. Some contemporary light curing units now feature high-irradiance, short light-activation modes lasting only 1 to 5 s, which could reduce treatment duration—an important advantage when using them in pediatric dentistry [15,16].

Although colored compomers are widely utilized in pediatric clinical practice, the current literature lacks comprehensive data on how color shade and curing protocols (in terms of both time and light intensity) influence their polymerization efficiency and mechanical behavior. Specifically, the interplay between the DC and microhardness across various color shades under different light curing strategies has not been sufficiently addressed. Moreover, color-dependent differences in clinical behavior—such as susceptibility to undercuring in darker shades—remain underexplored.

This study aims to fill a critical gap in the current literature by systematically evaluating the influence of color shade and curing duration on the degree of conversion and microhardness of colored compomers. Utilizing fourier transform infrared (FTIR) spectroscopy and Vickers microhardness testing, it provides a comprehensive analysis of both the chemical and mechanical aspects of polymerization. Three distinct light curing protocols—ultra-fast (3 s/3200 mW/cm^2^), intermediate (10 s/1000 mW/cm^2^), and conventional (20 s/1000 mW/cm^2^)—were employed to reflect current clinical practices. The outcomes of this research can inform the development of evidence-based, color-specific curing guidelines and contribute to the optimization of restorative protocols in pediatric dentistry, ultimately improving material performance and patient compliance.

## 2. Materials and Methods

In this study, eight different-colored commercial resin compomers (gold, blackberry, green, pink, orange, lemon, blue, and silver) (Twinky Star, VOCO, Cuxhaven, Germany) were used as study groups, while an A2 compomer (Glasiosite VOCO, Cuxhaven, Germany) was used as a control. Some technical properties and commercial and compositional information of the restorative materials and contents used in the study are given in Table 1.

### 2.1. Sample Preparation

The sample size was determined based on a power analysis carried out using G*Power 3.1 (Heinrich Heine University, Düsseldorf, Germany). To detect a medium effect size (f = 0.25) with 80% power at a 5% significance level in a two-way ANOVA design (color × curing time), a minimum of 81 samples was required (9 per group). This estimation ensured sufficient statistical power to identify significant differences among the experimental conditions with three curing times across nine color groups for both DC and microhardness evaluations.

A total 162 samples were prepared for both FTIR analysis (*n* = 81) and microhardness testing (*n* = 81) (9 samples per group). Each group was further divided into three subgroups based on the light curing times. Disk-shaped samples were prepared using polytetrafluoroethylene molds with a 5 mm diameter and 2 mm depth [16]. The compomers were placed into the molds with a mouth spatula and pressed with a flat glass plate. Each disk was then polymerized using a multi-wavelength light-emitting diode curing device (Valo™ Cordless, Ultradent Products Inc., South Jordan, UT, USA) at different curing times and light intensities (3 s/3200 mW/cm^2^, 10 s/1000 mW/cm^2^, 20 s/1000 mW/cm^2^). The curing device was tested with a radiometer (Proclinic, Zaragoza, Spain) before use. The upper surface of each sample was progressively polished with silicon carbide abrasive disks (Soft-Lex, 3M ESPE Dental Products, St. Paul, MN, USA) with different thicknesses (coarse, medium, light) for 20 s each. The samples were stored in distilled water at 37 °C in a dark environment until use.

### 2.2. Determination of Degree of Conversion

FTIR spectra of the samples were obtained in absorbance mode using a Nicolet iS10 FT-IR spectrometer (Thermo Fisher Scientific, Waltham, MA, USA), equipped with a Smart iTR ATR accessory containing a diamond crystal and operated with OMNIC software (Omnic Driver version:8.2). Measurements were conducted within the wavenumber range of 525–4000 cm^−1^ at a resolution of 8 cm^−1^, with 32 scans per sample. The acquired spectra were analyzed to assess the degree of curing and quantify crosslinking reactions under different curing conditions, utilizing Origin pro software (Origin pro 2019b, 9.6.5.169). The calculation of the curing degree was based on the characteristic absorption peak areas, following the equation provided below.

$$Conversion\;Degree\left(\%\right)=\{1-(\frac{(AP\;1636/AP\;1604)psc}{(AP\;1636/AP\;1604)prc})\}\;\times \;100\%$$

**Figure 1:**
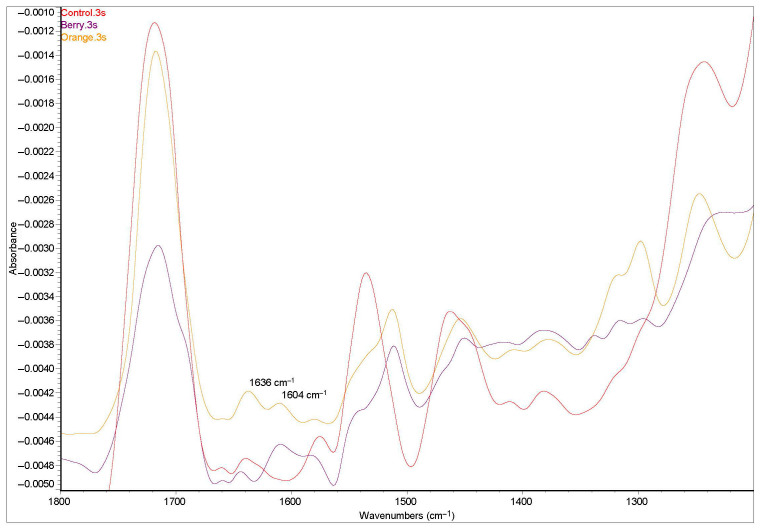
Representative FTIR spectra demonstrating the maximum and minimum degrees of conversion observed in the colored compomers and control group after a 3 s polymerization process.

According to this equation, the characteristic absorption peaks were at 1636 cm^−1^ (unsaturated aliphatic C=C double bonds originating from methacrylate groups) and 1604 cm^−1^ (aromatic C=C double bonds) [17]. The term “AP” represents the intensity of a specific absorption peak, where “psc” refers to “post-curing” and “prc” refers to “pre-curing.” The absorbance peak examples at wavelengths 1636 cm^−1^ and 1604 cm^−1^ following 3 s polymerization are shown in Figure 1.

### 2.3. Determination of Microhardness

The Vickers hardness values were measured from the top surfaces of a total of 81 samples, which were polished using 400, 800, 1000, 1500, 2000, and 2500 grit silicon carbide papers. Then, five indentations, with a constant load of 200 g for 15 s [18], were inflicted on each surface, one in the center and one in each quadrant (>100 μm from each other). The average of the results was reported as the Vickers hardness value.

### 2.4. Statistical Analysis

Statistical analyses were performed using Jamovi 2.6.26, with continuous variables summarized via descriptive statistics. Normality was assessed using Shapiro–Wilk tests and Q-Q plots, with non-normal data subjected to Box–Cox transformations. A two-way ANOVA examined the effects of color and curing time (including interaction effects), and this was followed by Tukey’s HSD post hoc tests (α = 0.05). Spearman’s correlation (ρ) evaluated the relationship between the DC and microhardness.

## 3. Results

The chemical and mechanical properties of colored compomers are profoundly influenced by their pigment composition and curing protocols, which directly determine their clinical success. This study systematically evaluates the DC and microhardness of compomers across a spectrum of colors and curing durations, uncovering significant interactions between these factors.

### 3.1. DC Results 

Table 2 presents the comparative analysis of the DC values of the colored compomers against those of the control group, regardless of curing time. In terms of the DC, all the colored groups showed significantly lower performance compared to the control group (*p* < 0.001), with green compomers recording the lowest DC values. Inter-color comparisons revealed that the green group exhibited significantly lower DC values than the orange (t = −4.47, *p* = 0.001) and gold (t = 3.30, *p* = 0.041) compomers. Statistical analysis with multiple comparisons revealed that the green compomers exhibited significantly lower DC values compared to the orange (*p* = 0.001) and gold (*p* = 0.041) compomers. No significant differences were observed among the blue, gold, lemon, and pink compomers (*p* > 0.05) in the post hoc tests.

The effect of curing time on the DC of the colored compomers, regardless of their color, is systematically analyzed in Table 3. The statistical analysis revealed significant effects of curing time on the degree of conversion (*p* < 0.05). The 10 s curing time demonstrated significantly lower DC values compared to both 3 s (*p* = 0.037) and 20 s (*p* = 0.005) durations, while no significant difference was observed between the 3 s and 20 s groups (*p* = 0.735).

The two-way ANOVA revealed a statistically significant interaction between color and curing time (F(16) = 3.35, *p* < 0.001), indicating that the effect of curing time on the DC was dependent on material color (Table 4). Notably, orange compomers exhibited the highest DC values at 3 s (73.1 ± 8.58), which were comparable to those of the control group (74.3 ± 1.19, *p* = 0.456), but showed a dramatic decrease at 10 s (36.3 ± 0.16, *p* = 0.005). The green and berry shades consistently demonstrated the lowest DC values across all curing times (all comparisons with control *p* < 0.01). The inter-color analysis revealed significant variations in polymerization efficiency among the different shades when compared at identical curing durations. At the 3 s interval, the orange compomers demonstrated superior degree of conversion values compared to all the other colored groups (*p* < 0.05), while lemon shades significantly outperformed berry, green, pink, and silver materials (*p* < 0.05). The control group maintained its expected dominance over all pigmented compomers at this duration (*p* < 0.001). For 10 s curing, the gold compomers showed better conversion than the green shades (*p* = 0.047), and silver exceeded berry (*p* = 0.039), though all colored groups remained inferior to the control (*p* < 0.001). The 20 s comparison showed that blue compomers achieved higher conversion than both the green and lemon shades (*p* < 0.05), with pink compomers similarly surpassing these two shades (*p* < 0.01).

### 3.2. Microhardness Results

A comparative analysis of the microhardness values of the colored compomers, regardless of curing time, is given Table 5. The silver and control groups demonstrated the highest values, while the gold compomers showed significantly lower hardness compared to the silver (t = 5.78, *p* < 0.001) and orange (t = 4.21, *p* = 0.002) groups.

Multiple-comparison analysis revealed that the gold compomers exhibited significantly lower values compared to both the silver (*p* < 0.001) and orange (*p* = 0.002) compomers. Meanwhile, the pink and blue compomers showed intermediate values, with no statistically significant differences between them (*p* > 0.05).

The effect of curing time on the microhardness of the colored compomers, regardless of their color, is systematically analyzed in Table 6. Microhardness was significantly influenced by curing time (*p* = 0.042). The 20 s curing duration produced the highest microhardness values (93.5 ± 48.2), which were significantly greater than those achieved with 10 s curing (83.3 ± 36.9; *p* = 0.041). However, no significant difference was found between the 3 s (87.1 ± 28.4) and 20 s curing durations (*p* > 0.05).

The statistical analysis revealed a significant effect of interactions between compomer color and curing time on microhardness (*p* < 0.05) (Table 7). The pink compomers exhibited exceptional initial microhardness at 3 s (134.0 ± 0.0) that progressively deteriorated with extended curing (*p* < 0.001), while the blue compomers showed particular vulnerability at 10 s (53.7 ± 21.2) compared to other durations (*p* = 0.018). The gold compomers displayed substantial variability at 20 s (130.3 ± 113.1), despite exhibiting high mean values, indicating polymerization inconsistency. The control samples maintained superior and stable microhardness across durations (*p* < 0.05 versus most colored compomers), except when compared to pink at 3 s and gold at 20 s. The 10 s curing duration emerged as particularly detrimental for the blue, green, and lemon groups, while 3 s curing optimized performance for the pink compomers and controls. The inter-color comparative analysis revealed significant variations in microhardness performance among differently pigmented compomers at identical curing durations. At the 3 s interval, the pink compomers demonstrated exceptional mechanical properties (134.0 ± 0.0), significantly surpassing all other materials (*p* < 0.001), while the control samples maintained their expected superiority over the gold, green, and lemon groups (*p* < 0.05). Blue compomers after the 10 s curing duration showed the lowest microhardness values (53.7 ± 21.2) and were significantly outperformed by both the silver compomers (102.0 ± 18.2, *p* < 0.01) and the control samples (*p* < 0.001). Notably, the gold compomers exhibited a unique pattern at 20 s curing, achieving the highest mean microhardness (130.3 ± 113.1), but with substantial variance that limited their statistical superiority to being relative only to the blue and lemon groups (*p* < 0.05).

### 3.3. Relationship Between DC and Microhardness Values

**Figure 2:**
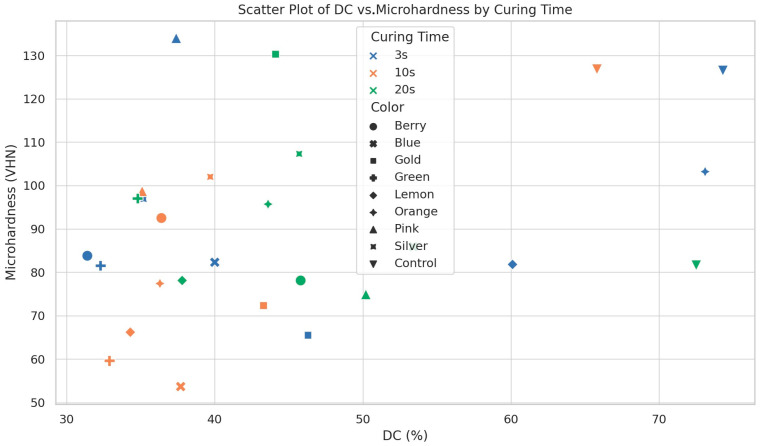
A scatter plot showing the relationship between the DC and microhardness of the colored compomers at different curing times. Each point represents the average value for a specific color and curing duration.

The relationship between the DC and the microhardness values of the colored compomers was separately analyzed for each color group and polymerization time using Spearman correlation analysis (Figure 2). The results demonstrate that the DC–microhardness relationship varies significantly depending on both color and curing time. While a moderate positive correlation (ρ = 0.42, *p* = 0.003) was observed for the entire dataset, distinct differences emerged among color groups. For instance, the orange compomers showed a strong positive correlation (ρ = 0.78, *p* = 0.001), whereas the pink compomers exhibited no significant relationship (ρ = −0.15, *p* = 0.512). Darker colors like green and blue demonstrated moderate positive correlations, though their absolute DC and microhardness values remained low. Time-dependent analysis revealed significant positive correlations at the 3 s (ρ = 0.51, *p* = 0.010) and 20 s (ρ = 0.63, *p* = 0.002) curing times, but not at 10 s (ρ = 0.18, *p* = 0.380). A negative correlation was found for the gold compomers (ρ = −0.41, *p* = 0.054).

## 4. Discussion

The present study highlights the significant influence of pigment chemistry and curing parameters on the polymerization kinetics and mechanical properties of dental compomers, with notable variations observed across different colors. The results demonstrate that the colored compomers exhibited a markedly lower DC compared to the control group, with green compomers showing the lowest DC (33.3%) and orange compomers showing the highest (51.0%). These observations align with previous research indicating that pigmentation and filler content can interfere with light penetration, thereby affecting polymerization depth and efficiency [18,19]. Green compomers, in particular, demonstrated consistently low DC values across all curing times, reaching only 33.3% on average. This finding parallels the report by Gönül & Kılıç [20], who found green and blue Twinky Star shades to require longer curing durations to reach acceptable microhardness. Similarly, Magalhães et al. [21] showed that green and lemon shades are more susceptible to surface degradation under erosive–abrasive conditions, likely due to incomplete polymerization and lower crosslink density. In our study, these shades also exhibited poor mechanical performance, reinforcing the notion that insufficient light transmission results in undercured and structurally compromised restorations.

The observed color-dependent variations in the DC can be attributed to several physicochemical mechanisms. Darker pigments, particularly in green and berry compomers, likely absorb a significant portion of the curing light’s energy, thereby reducing the effective photon density available for initiating the polymerization reaction [10]. This phenomenon is exacerbated by the presence of glitter particles in these materials, which may create light-scattering effects [22]. This substantial discrepancy warrants careful consideration in clinical applications, particularly in pediatric dentistry, where these materials are frequently employed for their psychological benefits. The aesthetic appeal of colored compomers must be balanced with their clinical performance [7,23].

In contrast, orange compomers showed a surprising outcome. When cured for 3 s at high intensity (3200 mW/cm^2^), the orange group achieved a DC of 73.1%, nearly matching the control group (74.3%), with no statistically significant difference (*p* = 0.456). This exceptional performance may be linked to the spectral overlap between the orange pigments and the emission spectrum of the curing unit. Shimokawa et al. [24] emphasized that spectral matching between photoinitiator absorption and light source emission improves polymerization efficiency. In our case, it is plausible that the diketopyrrolopyrrole-based pigments used in the orange formulation exhibit partial spectral resonance with blue-light wavelengths, enhancing curing efficiency beyond that of many conventional shades. Such a mechanism would not only reduce light attenuation, but also potentially amplify photoinitiator activation due to better light absorption at target wavelengths.

The observed variations in the DC and microhardness can be further elucidated through the lens of polymer chemistry and structural dynamics. The degree of conversion is intrinsically linked to the crosslinking density of the polymer network, which is influenced by the mobility of methacrylate monomers during curing. Darker pigments (e.g., green) likely absorb or scatter curing light, reducing photon availability for photoinitiators and resulting in lower crosslink density and unreacted C=C bonds, as evidenced by FTIR. This incomplete polymerization compromises the mechanical integrity, explaining the reduced microhardness. Conversely, orange compomers’ superior performance under high-intensity curing suggests efficient light penetration and initiator activation, yielding a more homogeneous and densely crosslinked matrix. Additionally, filler–pigment interactions may alter stress distribution under indentation, contributing to color-dependent hardness variations. Thus, both chemical (monomer conversion) and physical (filler dispersion) factors collectively dictate the material’s mechanical behavior [18,19,24].

Microhardness values varied significantly across colors, with the gold compomers exhibiting the lowest values and the silver compomers performing comparably to the control group. Similarly to the study by Jafari et al. [25], which reported that silver compomers exhibited the highest microhardness while blue compomers showed the lowest, our results confirm that pigment composition significantly influences material performance. However, our study provides a more comprehensive analysis by incorporating DC measurements alongside microhardness, revealing that darker shades (e.g., green) not only demonstrate lower microhardness, but also a significantly reduced DC (33.3%), likely due to light absorption by pigments. This variability suggests that the mechanical properties of compomers are not solely dependent on the degree of conversion, but are also influenced by the intrinsic characteristics of the pigments and fillers used [13]. The moderate correlation between the DC and microhardness (ρ = 0.42) further supports the notion that while higher conversion rates generally improve mechanical properties, other factors, such as filler distribution and resin matrix composition, also play a role [19]. While the silver compomers achieved values comparable to those of the control (106.6 vs. 104.4 VHN), the gold compomers showed remarkably low and variable hardness (88.1 ± 72.1 VHN). This extreme variability suggests potential issues with filler distribution or particle–matrix adhesion in certain formulations [19]. The moderate overall correlation between the DC and microhardness (ρ = 0.42) indicates that while the conversion rate is an important factor, other material characteristics, such as filler type, size distribution, and resin matrix composition, play significant roles in determining mechanical properties [13].

The curing time experiments yielded particularly noteworthy findings. The 10 s protocol consistently produced inferior results for both the DC and microhardness across most colors, while the 3 s high-intensity and 20 s standard-intensity protocols showed comparable performance. This challenges the conventional assumption that intermediate curing times provide optimal results [26], and suggests that extremely short, high-intensity curing may be more effective for certain applications. The exceptional performance of orange compomers under 3 s curing (73.1% DC) approaches control values and indicates that some colored formulations may be particularly suited to rapid polymerization protocols.

In addition to light exposure parameters, post-curing protocols and curing mode selection can also significantly influence the polymerization efficiency and mechanical properties of dental materials. Karademir et al. [27] found that extending the post-curing duration enhanced both the degree of conversion and the microhardness of 3D-printed permanent resins, while reducing stainability. Similarly, İyibilir et al. [28] demonstrated that third-generation LED curing units operating in high-power or Xtra-power modes improved the surface hardness of certain composite resins, although material-specific responses were noted. These findings support our observation that curing efficiency and resultant mechanical behavior are multifactorial and strongly dependent on both the material composition and curing strategy.

The current findings significantly expand upon previous work by Khodadadi et al. [29] and Shimokawa et al. [24] regarding light curing of colored restorative materials. Our results confirm, but substantially refine, Khodadadi’s observation that colored compomers exhibit different hardness patterns compared to hybrid compomers, demonstrating that these differences are not merely material-dependent, but vary systematically with both pigment chemistry and curing parameters.

Our results align with recent investigations into colored compomer performance. Duruk et al. [30] reported higher residual monomer release (particularly BisGMA release) in gold-colored compomers, correlating with our observed lower microhardness in gold shades, suggesting incomplete polymerization. Notably, both studies found that darker pigments (e.g., green) underperformed, likely due to light absorption. While Duruk et al. [30] linked viscosity to monomer release, our data further demonstrate that high-intensity short curing may mitigate these effects in lighter shades (e.g., orange), emphasizing the need for color-specific protocols.

Clinicians should be aware that color choice affects not just aesthetics, but also material performance. Lighter colors like orange and silver may offer better mechanical properties than darker shades. The traditional 20 s curing time may not be necessary for all colors, potentially reducing chair time in pediatric cases. However, darker shades still require extended curing. The lower DC observed in colored compomers raises concerns about increased monomer leaching and potential biological effects [9], suggesting the need for careful follow-up of these restorations. In line with these concerns, Bezgin et al. [31] reported that pediatric restorative materials such as compomers and glass ionomer cements can release various residual monomers—including HEMA, Bis-GMA, and TEGDMA—especially during the early post-polymerization period, potentially causing cytotoxic or allergenic responses. This highlights the importance of optimizing curing protocols to minimize residual monomer release, particularly for darker shades with a lower DC.

This study has several limitations. First, only one brand of colored compomer (Twinky Star, VOCO) was investigated. Although this product line is widely used in pediatric dentistry, the results may not be generalizable to other formulations with different pigment compositions or filler technologies. Additionally, the in vitro setting of this study does not fully replicate clinical conditions, where variables such as intraoral temperature, access limitations, and operator technique may further influence curing efficiency. While our study varied both the time and intensity concurrently, future work should isolate light intensity effects by testing fixed durations across multiple power densities. This would clarify whether high-intensity curing alone can mitigate pigment-related light attenuation, particularly in darker shades like green or berry, where the DC was consistently low, regardless of duration.

## 5. Conclusions

This comprehensive investigation reveals that colored compomers exhibit distinct polymerization behaviors and mechanical properties that are significantly influenced by both their chromatic characteristics and curing parameters. While these materials fulfill an important psychological role in pediatric dentistry, their technical performance varies substantially from that of conventional compomers. The study demonstrates the following: Color selection directly impacts material properties, with darker shades generally showing poorer conversion and mechanical performance. Curing protocols should be color-specific, with high-intensity short curing suitable for lighter shades, but extended times required for darker pigments. The relationship between the degree of conversion and microhardness is complex and color-dependent, indicating that material formulation plays a crucial role in determining clinical performance.

These findings can guide both clinical practice and future material development. Manufacturers might consider reformulating darker shades to improve light transmission, while clinicians should adapt curing protocols based on restoration color. Further research should investigate the long-term clinical performance of these materials and explore potential modifications to enhance their physicochemical properties without compromising their aesthetic appeal.

## Figures and Tables

**Table 1:** Materials used in the study.

Material	Color	Manufacturer	Ingredients
Twinky Star	Berry Lemon Green Silver Blue Pink Gold Orange	Voco, Cuxhaven, Germany	Ba-AI-Str-fluorosilicate glass, silicon dioxide, Bis-GMA, UDMA, carboxylic acid modified methacrylate, kamforkinon, BHT
Glasiosite	A2	Voco, Cuxhaven, Germany	UDMA, Bis-GMA mixture of diff. dimethacrylates, glass ceramics, silicates, initiators

**Table 2:** Comparative analysis of the DC values of the colored compomers, regardless of curing time.

Color	*n*	Median (Min–Max)	Test Statistic	*p*-Value
Berry	9	37.5 (23.0–54.9) ^de^	H(8) = 31.19	*p* < 0.05
Blue	9	39.8 (37.1–69.4) ^cd^		
Gold	9	43.4 (33.2–62.3) ^bc^		
Green	9	32.1 (26.5–46.9) ^e^		
Lemon	9	38.0 (28.8–67.5) ^cd^		
Orange	9	44.3 (36.0–89.6) ^ab^		
Pink	9	36.5 (34.9–54.0) ^d^		
Silver	9	42.4 (13.4–49.5) ^bc^		
Control	9	71.7 (62.5–76.7) ^a^		

Non-parametric tests (Kruskal–Wallis/Mann–Whitney) were used due to non-normal distribution. Different superscript lowercase letters indicate statistical significance (*p* < 0.05).

**Table 3:** Comparative analysis of the DC values of the colored compomers, regardless of color.

Curing Time	*n*	Mean ± SD	Test Statistic	*p*-Value
3 s	27	47.8 ± 18.4 ^ac^	F(2) = 5.93	*p* < 0.05
10 s	27	40.2 ± 9.9 ^b^		
20 s	27	47.5 ± 11.9 ^c^		

Parametric testing (ANOVA) was used for normally distributed data. Different superscript lowercase letters indicate statistical significance (*p* < 0.05).

**Table 4:** DC values by color and interaction with curing time, with statistical comparisons.

Color	3 s (Mean ± SE)	10 s (Mean ± SE)	20 s (Mean ± SE)	*p*-Value
Berry	31.4 ± 4.36 ^e,B^	36.4 ± 0.68 ^de,AB^	45.8 ± 4.54 ^cd,A^	0.018
Blue	40.0 ± 0.26 ^de,AB^	37.7 ± 0.30 ^e,B^	53.4 ± 8.15 ^abc,A^	0.042
Gold	46.3 ± 8.51 ^bcd,A^	43.3 ± 0.07 ^cd,A^	44.1 ± 0.88 ^cd,A^	>0.05
Green	32.3 ± 0.57 ^e,A^	32.9 ± 2.18 ^e,A^	34.8 ± 6.19 ^e,A^	>0.05
Lemon	60.1 ± 3.75 ^ab,A^	34.3 ± 2.80 ^e,B^	37.8 ± 0.31 ^de,B^	0.012
Orange	73.1 ± 8.58 ^a,A^	36.3 ± 0.16 ^e,B^	43.6 ± 1.73 ^cd,B^	<0.05
Pink	37.4 ± 1.16 ^de,B^	35.1 ± 0.11 ^e,B^	50.2 ± 2.07 ^abc,A^	<0.05
Silver	35.2 ± 10.88 ^e,B^	39.7 ± 0.90 ^de,AB^	45.7 ± 2.08 ^cd,A^	0.049
Control	74.3 ± 1.19 ^a,A^	65.8 ± 2.48 ^a,B^	72.5 ± 0.86 ^a,A^	<0.05
***p*-Value**	<0.05	<0.05	<0.05	

Two-way ANOVA with Tukey’s HSD post hoc test was used for main effects and interactions (α = 0.05). For non-normal distributions (green, pink groups), the Kruskal–Wallis test with Dunn–Bonferroni correction was applied. Different superscript lowercase letters in each column indicate statistical significance (*p* < 0.05). Different superscript uppercase letters in each row indicate statistical significance (*p* < 0.05).

**Table 5:** Comparative analysis of the microhardness values of the colored compomers, regardless of curing time.

Color	*n*	Median (Min–Max)	Test Statistic	*p*-Value
Berry	9	70.8 (66.6–136.1] ^c^	H(8) = 10.8	<0.001
Blue	9	77.5 (35.3–112.2] ^bc^		
Gold	9	83.5 (31.2–261.6] ^bc^		
Green	9	79.5 (40.6–115.8] ^bc^		
Lemon	9	73.5 (59.8–88.6] ^c^		
Orange	9	74.0 (51.0–152.6] ^bc^		
Pink	9	77.2 (74.9–134.0] ^ab^		
Silver	9	92.6 (71.9–166.8] ^a^		
Control	9	94.9 (70.5–199.7] ^a^		

Non-parametric testing was used due to skewed non-normal distributions. Different superscript lowercase letters indicate statistical significance (*p* < 0.05).

**Table 6:** Comparative analysis of the microhardness values of the colored compomers, regardless of color.

Curing Time	*n*	Mean ± SD	Test Statistic (ANOVA)	*p*-Value
3 s	27	87.1 ± 28.4 ^ab^	F(2) = 3.42	0.042 *
10 s	27	83.3 ± 36.9 ^a^		
20 s	27	93.5 ± 48.2 ^b^		

Parametric testing (ANOVA) was used for normally distributed data. Different superscript lowercase letters indicate statistical significance. * *p* < 0.05 is statistically significant

**Table 7:** Microhardness values by color and curing time, with statistical comparisons.

Color	3 s (Mean ± SE)	10 s (Mean ± SE)	20 s (Mean ± SE)	*p*-Value
Berry	83.8 ± 16.8 ^cd,A^	92.5 ± 34.6 ^ab,A^	78.1 ± 15.1 ^bc,A^	0.451
Blue	82.3 ± 33.0 ^cd,AB^	53.7 ± 21.2 ^e,B^	85.9 ± 21.1 ^abc,A^	0.024
Gold	65.5 ± 26.4 ^d,A^	72.3 ± 13.3 ^cd,A^	130.3 ± 113.1 ^a,A^	0.089
Green	81.5 ± 24.2 ^cd,AB^	59.6 ± 23.5 ^de,B^	97.0 ± 18.2 ^ab,A^	0.020
Lemon	81.8 ± 8.0 ^cd,A^	66.2 ± 6.8 ^de,B^	78.1 ± 8.8 ^c,AB^	0.004
Orange	103.2 ± 39.6 ^abc,A^	77.4 ± 28.2 ^bc,B^	95.7 ± 47.0 ^ab,AB^	0.210
Pink	134.0 ± 0.0 ^a,A^	98.7 ± 0.0 ^ab,B^	74.9 ± 0.0 ^c,C^	0.001
Silver	96.9 ± 21.3 ^bc,A^	102.0 ± 18.2 ^a,A^	107.3 ± 44.6 ^a,A^	0.678
Control	126.5 ± 23.3 ^ab,A^	126.8 ± 57.4 ^a,A^	81.6 ± 12.5 ^abc,A^	0.123
***p*-Value**	<0.001	<0.001	<0.05	

The data were analyzed using Welch–ANOVA with Games–Howell post hoc tests (for non-normal distributions: gold, blue, lemon; Shapiro–Wilk *p* < 0.05) and the Kruskal–Wallis test with adjusted post hoc tests (for unequal variances; Levene’s *p* = 0.008). Different superscript lowercase letters in each column indicate statistical significance (*p* < 0.05). Different superscript uppercase letters in each row indicate statistical significance (*p* < 0.05).

## Data Availability

The original contributions presented in this study are included in the article. Further inquiries can be directed to the corresponding author.

## Abbreviations

The following abbreviations are used in this manuscript:

DC	Degree of conversion
FTIR	Fourier transform infrared spectroscopy
